# Open Aortic Repair After Thoracic Endovascular Aortic Repair: Strategic Insights From a Single Centre Surgical Experience

**DOI:** 10.1016/j.ejvsvf.2024.12.001

**Published:** 2024-12-31

**Authors:** Yutaka Iba, Tomohiro Nakajima, Junji Nakazawa, Tsuyoshi Shibata, Shuhei Miura, Nobuyoshi Kawaharada

**Affiliations:** Department of Cardiovascular Surgery, Sapporo Medical University, Sapporo, Hokkaido, Japan

**Keywords:** Aortic Aneurysm, Aortic Dissection, Complication, Thoracic Endovascular Aneurysm Repair

## Abstract

**Objective:**

Thoracic endovascular aortic repair (TEVAR) is widely used in thoracic aortic surgery. However, for various reasons some patients require secondary open aortic repair. Herein, the surgical outcomes and problems of such open conversion surgery after TEVAR are investigated.

**Methods:**

This was a retrospective and observational study. From January 2010 to June 2022, 20 patients who underwent open aortic repair after TEVAR were included. The indications for open conversion surgery were as follows: aortic enlargement due to endoleak (EL) in seven patients (type Ia: *n* = 4, type II: *n* = 1, type V: *n* = 2; 35%), stent graft infection including aorto-oesophageal fistula (AEF) in six (30%), retrograde type A aortic dissection (RTAD) in three (15%), and dilatation of adjacent distal aorta or false lumen in four (20%).

**Results:**

Seven patients with type Ia EL or RTAD required open aortic arch repair. Four underwent thoraco-abdominal aortic repair for distal aortic enlargement. Descending thoracic aortic replacement was performed in all six infection cases and two patients with type V EL. Furthermore, three patients with AEF received concomitant oesophagectomy. One patient with persistent type II EL underwent intercostal artery ligation and aneurysmorrhaphy via thoracotomy. There were two in hospital deaths (10%), all with AEF. Thus, the rates of in hospital death were 0% in non-infected cases, 33% in graft infections, with 66% in those with AEF. Stroke and paraplegia were observed in two patients (10%).

**Conclusion:**

When open conversion surgery is required after TEVAR, the indications are complex, often associated with infectious pathology, and are necessarily high risk particularly in patients with AEF. Surgical strategy has to be individualised based on the nature or cause of the complication and extent of aortic involvement.

## Introduction

Since the advent of thoracic endovascular aortic repair (TEVAR) as a minimally invasive aortic treatment option, it has been used increasingly over the years and has become the treatment of choice for thoracic aortic lesions with advances in its technology.^1^ However, some patients require secondary open aortic surgery because of new adjacent aortic pathologies or complications after TEVAR during long term follow up. According to a previous report, late open surgical conversions after TEVAR are observed in 3–8% of cases.^2^, ^3^, ^4^, ^5^, ^6^ Such secondary operations require a specific surgical strategy unlike conventional aortic re-operation and particularly raise the issue of how to deal with the endograft, such as preserving it, extracting it, or cutting only part of it off.^7^ In this study, experiences with open surgical conversion after TEVAR are reported, including the relationship between its causes and surgical procedures and investigate the surgical outcomes and problems of such open conversion surgery after TEVAR.

## Material and methods

Between January 2010 and June 2022, 20 patients underwent open aortic repair after TEVAR. These patients were a consecutive series from a single institution and were investigated retrospectively. Patients who underwent prior TEVAR in other hospitals and were referred to the centre after developing complications were also included. The Institutional Review Board of Sapporo Medical University Hospital approved this retrospective study (342-214) and waived the requirement of individual patient consent because of its retrospective nature.

### Operative procedure

**Figure 1:**
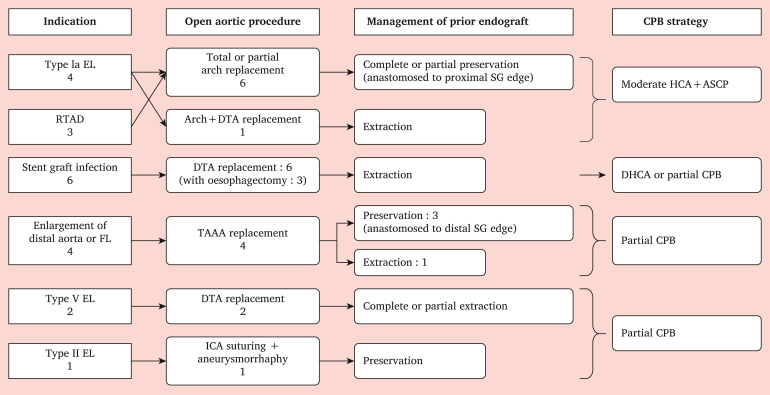
Surgical strategy for open conversion after thoracic endovascular aortic repair including extracorporeal circulation and management of prior stent grafts based on individual aetiology. EL = endoleak; RTAD = retrograde type A aortic dissection; FL = false lumen; ICA = internal carotid artery; SG = stent graft; HCA = hypothermic circulatory arrest; DHCA = deep hypothermic circulatory arrest; CPB = cardiopulmonary bypass; DTA = descending thoracic aorta; TAAA = thoraco-abdominal aorta aneurysm.

Surgical procedures including management of prior stent grafts and strategies for extracorporeal circulation for each indication are summarised in Figure 1.

### Type Ia endoleak and retrograde type A aortic dissection

**Figure 2:**
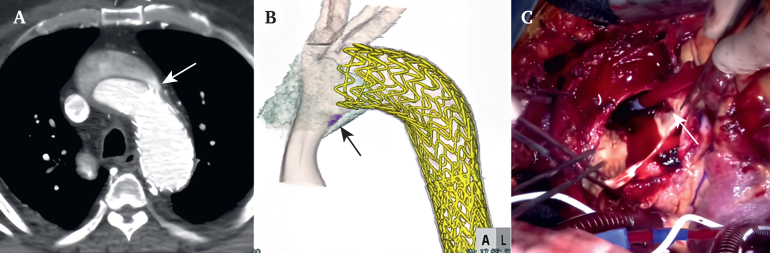
Pre-operative examination and operative findings in retrograde type A aortic dissection cases. (A,B) Pre-operative computed tomography shows an intimal tear (arrow) adjacent to the proximal end of the stent graft. (C) Intra-operative findings also show an intimal tear on the lesser curvature of the arch close to the proximal end of the stent graft (arrow).

For patients with type Ia endoleak or retrograde type A aortic dissection (RTAD), proximal aortic surgery is usually required (Fig. 2). In such a situation, median sternotomy is performed, and conventional cardiopulmonary bypass (CPB) is established with ascending aortic cannulation and right atrial drainage. Aortic arch replacement is then performed using moderate hypothermic circulatory arrest and antegrade selective cerebral perfusion (ASCP). At this time, either the arch prosthesis is anastomosed to the edge of the stent graft and aortic wall, or in some cases, the stent graft is partially cut off or the bare part of it is excised to make the stump for distal anastomosis. One patient underwent aortic arch and descending aortic replacement through a median sternotomy and left thoracotomy because chronic type B aortic dissection was primarily complicated.

### Infection cases

**Figure 3:**
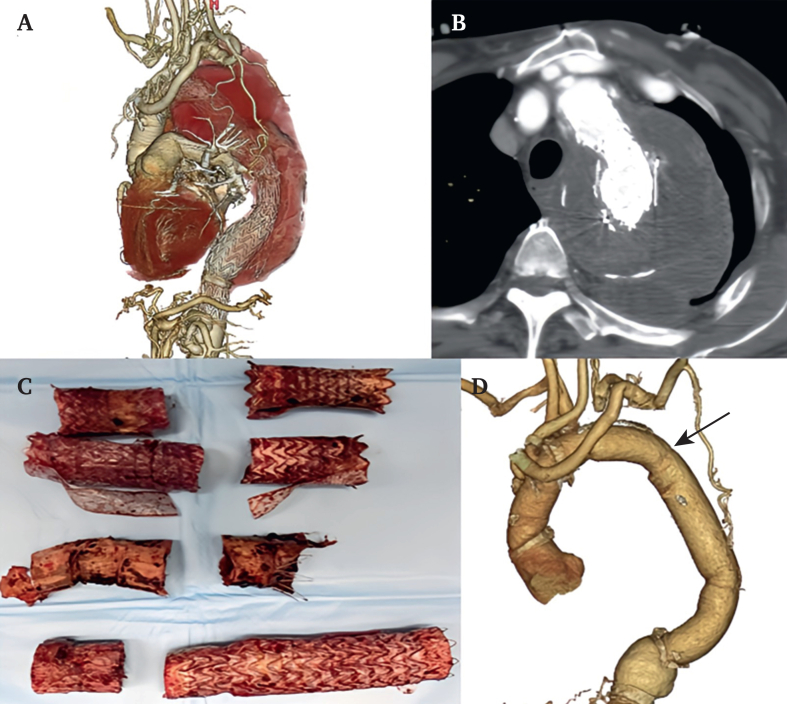
Pre- and post-operative examinations in cases of type V endoleak. (A, B) Pre-operative computed tomography (CT) shows an enlarged aortic aneurysm up to 10 cm despite multiple stent graft procedures. (C) Extracted stent grafts including cuffs in this case. (D) CT demonstrates the reconstructed aorta with prosthetic graft following complete extraction of the stent graft (arrow). Anastomosis of a prior elephant trunk to a new prosthetic graft.

The basic surgical strategy for infected aortic lesions is to remove the prior stent graft and replace the descending thoracic aorta (DTA) with a new vascular prosthesis. Omentopexy or muscle flap coverage is performed simultaneously or as early staged surgery to prevent the recurrence of infection of the new prosthesis. Rifampicin soaked vascular grafts were used when available, and were used in four of the six infected cases. In three cases in which a stent graft was placed from the aortic arch or just below the left subclavian artery, deep hypothermic circulatory arrest (DHCA) was introduced to remove the stent graft, and partial arch or DTA replacement was performed using open proximal anastomosis. Other patients underwent descending aortic surgery with partial CPB via femorofemoral cannulation. Oesophagostomy was performed in all three patients with aorto-oesophageal fistula (AEF). The current strategy for AEF is to transect the oesophagus thoracoscopically through a right thorax approach in advance and then shift to a left thoracotomy and remove the oesophagus together with the infected aortic wall (Fig. 3). In the case of aorto-bronchial fistula, the aortic wall adjacent to the lung was removed as much as possible; however, lobectomy was not performed, and the surface of the lung in which a fistula was suspected was covered with the omentum.

### Distal aortic dilatation of false lumen enlargement

Thoracoabdominal aortic aneurysm (TAAA) repair is required for distal aortic dilatation or false lumen enlargement in aortic dissection cases after TEVAR. Currently, the straight incision with rib cross (SIRC) approach rather than the spiral incision is used to reach the TAAA to preserve the collaterals to the spinal cord, and then partial CPB is established with femorofemoral cannulation.^8^ The stent graft placed in the DTA is preserved and clamped with the native aortic wall, and the aorta is trimmed to the distal edge of the stent graft. Then, the stent graft is anastomosed to the prosthetic graft for TAAA replacement with the native aortic wall.

### Type V endoleak

**Figure 4:**
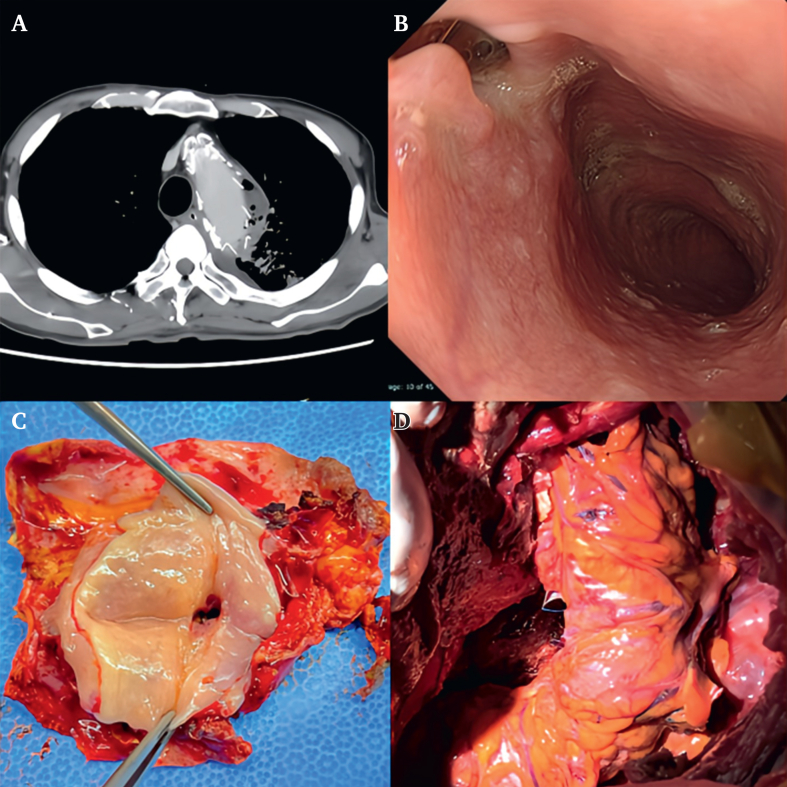
Pre-operative examination and operative findings in cases of aorto-oesophageal fistula. (A) Computed tomography reveals an air image in the aortic aneurysm outside the stent graft adjacent to the oesophagus. (B) Endoscopic findings show an oesophageal perforation and a stent beyond it. (C) Oesophageal perforation is observed in a specimen that was removed with the aortic wall. (D) The newly reconstructed prosthetic graft is finally wrapped with the omentum.

In cases where the descending aorta continues to expand even after TEVAR and no endoleaks can be identified on imaging surveillance, open descending aortic surgery may be unavoidable. In one patient, stent graft removal and descending aortic replacement were performed through left thoracotomy under partial CPB because the proximal descending aorta could be clamped (Fig. 4).

### Type II endoleak

In one patient, the DTA aneurysm continued to dilate because of blood inflow from the intercostal artery even after TEVAR. Therefore, the aneurysm was incised via left thoracotomy under partial CPB, the patent intercostal arteries were closed by sutures, and the aneurysmal wall was plicated as much as possible while preserving the stent graft.

### Statistical analyses

Statistical analyses were performed using JMP (version 17.0; SAS Institute, Inc., Cary, NC, USA). Continuous variables were evaluated for normality using the Shapiro–Wilk test and presented as means ± standard deviations if they followed a normal distribution and as medians and interquartile ranges if they did not. Categorical variables are presented as frequencies and percentages. Cumulative survival and freedom from aortic re-intervention rates were calculated using the Kaplan–Meier method.

## Results

The pre-operative characteristics of the patients are shown in Table 1. The reasons for open conversion surgery after TEVAR varied. Indications for open conversion surgery were as follows: aortic enlargement due to endoleaks (*n* = 7 patients, 35%) (type Ia: *n* = 4, type II: *n* = 1, and type V: *n* = 2), stent graft infection including AEF (*n* = 6 patients, 30%), RTAD (*n* = 3 patients, 15%), and dilatation of the adjacent distal aorta (*n* = 4 patients, 20%). In two type V cases, the aorta continued to enlarge to 90 and 106 mm in diameter, respectively, and a sealed rupture was suspected. Therefore, open conversion surgery was indicated. A patient with type II endoleak underwent open conversion surgery due to failure of coil embolisation for persistent aneurysm enlargement. In the three AEF cases, *Staphylococcus lugdunesis*, *Streptococcus oralis*, and *Corynebacterium* were detected in blood culture, respectively. Of the four cases that needed surgery for distal aorta, three cases of aortic dissection had enlarged false lumens because of blood flow from distal re-entry and one case of degenerative aneurysm had a large diameter 40 mm endograft used at the previous TEVAR. All cases of distal aortic enlargement were accompanied by thoraco-abdominal aortic lesions involving the visceral branches, therefore further endovascular treatment was unsuitable. Regarding the indications for pre-operative cerebrospinal fluid drainage (CSFD) in the repair of DTA and TAAA, conditions under antithrombotic therapy in urgent situations were excluded due to prior mechanical valve replacement, coronary intervention, or infection. The average number of previous thoracic aortic procedures, including endovascular and open repair, was 2.0 ± 1.2 for the 20 patients enrolled in this study. The mean interval from the previous aortic intervention to open conversion surgery was 34.0 ± 33.5 months. Follow up was complete for all patients. Mean follow up was 41.7 ± 42.3 months (maximum 142 months).

**Table 1:** Pre-operative patient (*n* = 20) characteristics.

Characteristics	Patients (*n* = 20)
Age – y	65 ± 11
Male sex	19 (95)
Hypertension	18 (90)
Diabetes	3 (15)
Prior cerebrovascular disease	3 (15)
Low cardiac function (LVEF <40%)	1 (5)
Coronary artery disease	3 (15)
COPD	3 (15)
CKD (eGFR <60 mL/min/1.73 m^2^)	10 (50)
Haemodialysis	1 (5)
Peripheral arterial disease	7 (35)
*Primary aetiology*	
Degenerative	10 (50)
Dissection	10 (50)
*Indication for open conversion*	
*Endoleak*	7 (35)
Ia	4
II	1
V	2
RTAD	3 (15)
Distal aortic dilatation	4 (20)
*Infection*	6 (30)
AEF	3
ABF	2
Connective tissue disorder	2 (10)
Maximum aortic diameter – mm	60 (52–69)
History of thoracic aortic surgery, times	2 (1–2)
Interval from first TEVAR to open conversion, months	21 (3–60)
Emergency operation	4 (20)

Continuous data are expressed as mean ± standard deviation or median (interquartile range), and categorial data as the *n* (%). LVEF = left ventricular ejection fraction; COPD = chronic obstructive pulmonary disease; CKD = chronic kidney disease; eGFR = estimated glomerular filtration rate; RTAD = retrograde type A aortic dissection; AEF = aorto-oesophageal fistula; ABF = aortobronchial fistula; TEVAR = thoracic endovascular aortic repair.

### Early result

Early outcomes are presented in Table 2. There were two in hospital deaths (10%), all with AEF; the rates of in hospital death were 0% in non-infected cases, 33% in graft infection cases, and 66% in those with AEF. One patient with AEF died of sepsis due to recurrent infection 30 days after open conversion surgery. The patient had undergone TEVAR twice, and in the second TEVAR, a branched stent graft was used, which covered the thoraco-abdominal region. AEF then occurred one year after the second TEVAR. Therefore, open descending aortic replacement, stent graft removal, oesophagectomy, and latissimus dorsi muscle flap were performed; however, the stent graft could not be completely removed because the stent graft branched. Therefore, the persistent infection did not improve, and the patient died of sepsis. Another patient with AEF had a well controlled infection; however, the patient underwent cardiopulmonary resuscitation for a sudden dysrhythmia and died of hypoxic encephalopathy two months after the operation. Permanent neurological deficit occurred in two patients (10%), including the aforementioned patient with AEF, and all of whom underwent descending aortic replacement using DHCA. Four patients had spinal cord injury (SCI), including two cases of paraplegia and two cases of paraparesis. Patients with paraplegia underwent extensive aortic graft replacement from the ascending aorta to the terminal aorta by multiple aortic interventions until open conversion surgery. The relationship between the reason for open conversion, details of the pre-operative background, surgical procedure, and early outcomes are shown in Table 3 for each case. The heterogeneity of the procedures in this study makes it difficult to draw strict comparisons; however, the mean operative time was 497 ± 212 minutes, and mean CPB bypass time was 207 ± 76 minutes.

**Table 2:** Early results.

Characteristics	Patients (*n* = 20)
30 day death	1 (5)
In hospital death	2 (10)
PND	2 (10)
TND	1 (5)
*SCI*	
Paraparesis	2 (10)
Paraplegia	2 (10)
Re-entry for bleeding	6 (30)
Intubation time – h	40 (15–62)
Prolonged ventilation – >72 h	4 (20)
Length of ICU stay – d	3 (1–4)
In hospital days	46 ± 25

Continuous data are expressed as mean ± standard deviation or median (interquartile range), and categorial data as *n* (%). PND = permanent neurological deficit; TND = temporary neurological dysfunction; SCI = spinal cord injury; ICU = intensive care unit.

**Table 3:** Details of pre-operative background, surgical procedure and outcome for individual patients.

No.	Age	Sex	Primary aortic aetiology	Indication for open conversion	Interval from latest TEVAR – mo	Open procedure	Extracorporeal circulation strategy	Endograft management	Early outcomes
1	54	M	CTBAD	Type Ia endoleak	95	Graft replacement of aortic arch	HCA with ASCP	Preservation	Discharged alive
2	71	M	Aneurysm	RTAD	0	Graft replacement of aortic arch	HCA with ASCP	Partial preservation	Discharged alive, paraparesis
3	72	M	Aneurysm	Type Ia endoleak	81	Graft replacement of aortic arch	HCA with ASCP	Partial preservation	Discharged alive
4	70	M	Aneurysm	ABF	50	Graft replacement of DTA	Partial CPB	Removal	Discharged alive
5	44	M	ATBAD	Type Ia endoleak	72	Graft replacement of arch and DTA	HCA with ASCP	Removal	Discharged alive
6	80	M	Aneurysm	AEF	2	Graft replacement of DTA + oesophagectomy	Partial CPB	Removal	Discharged alive
7	53	M	ATAAD	Enlargement of distal aorta	3	Graft replacement of TAAA	Partial CPB	Removal	Discharged alive, paraparesis
8	78	F	Aneurysm	Type Ia endoleak	43	Graft replacement of aortic arch	HCA with ASCP	Partial preservation	Discharged alive
9	87	M	CTBAD	RTAD	4	Graft replacement of aortic arch	HCA with ASCP	Preservation	Discharged alive
10	53	M	CTBAD	Enlargement of distal aorta	24	Graft replacement of TAAA	Partial CPB	Preservation	Discharged alive
11	74	M	Aneurysm	Type V endoleak	88	Graft replacement of DTA	Partial CPB	Preservation	Discharged alive
12	60	M	CTBAD	Enlargement of distal aorta	56	Graft replacement of TAAA	Partial CPB	Preservation	Discharged alive
13	68	M	Aneurysm	AEF	10	Graft replacement of DTA and oesophagectomy	DHCA	Removal	Died, Sepsis, PND
14	59	M	Aneurysm	Enlargement of distal aorta	3	Graft replacement of TAAA	Partial CPB	Preservation	Discharged alive, paraplegia
15	72	M	Aneurysm	Type II endoleak	42	Suture closure of ICA and aneurysmorrhaphy	Partial CPB	Preservation	Discharged alive
16	58	M	ATBAD	RTAD	82	Graft replacement of aortic arch	HCA with ASCP	Preservation	Discharged alive
17	65	M	CTBAD	Type V endoleak	1	Graft replacement of DTA	Partial CPB	Removal	Discharged alive, Paraplegia
18	58	M	CTBAD	Stent graft infection	18	Graft replacement of DTA	DHCA	Removal	Discharged alive, PND
19	51	M	CTBAD	ABF	1	Graft replacement of DTA	Partial CPB	Removal	Discharged alive
20	78	M	Aneurysm	AEF	4	Graft replacement of DTA and oesophagectomy	DHCA	Removal	Died, dysrhythmia, TND

CTBAD = chronic type B aortic dissection; ATBAD = acute type B aortic dissection; ATAAD = acute type A aortic dissection; RTAD = retrograde type A aortic dissection; ABF = aortobronchial fistula; AEF = aorto-oesophageal fistula; DTA = descending thoracic aorta; TAAA = thoraco-abdominal aorta; ICA= intercostal artery; HCA= hypothermic circulatory arrest; ASCP = antegrade selective cerebral perfusion; DHCA = deep hypothermic circulatory arrest; CPB = cardiopulmonary bypass; PND = permanent neurological deficit; TND = temporary neurological dysfunction.

### Late result

**Figure 5:**
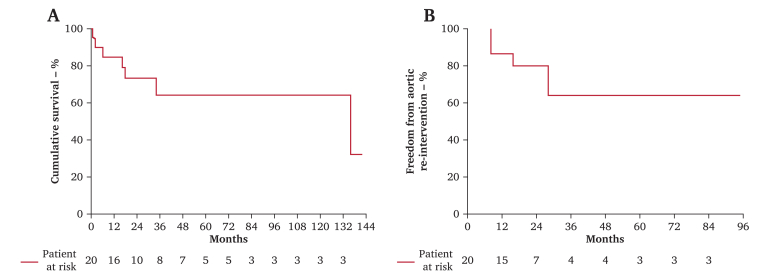
(A) Cumulative survival after open conversion surgery. (B) Freedom from aortic re-intervention after open conversion surgery.

Five late deaths were reported: two of heart failure, one of renal failure, one of cerebrovascular events, and one of respiratory failure; there were no aortic related deaths. The cumulative survival rate after open conversion surgery was 64.2% at five years and 64.2% at ten years. Late aortic re-intervention was required in four cases, all due to enlargement of the remaining distal aortic region, with three cases of TEVAR and one case of open abdominal aortic repair. The freedom from aortic re-intervention rate after open conversion surgery was 64.0% at five years, as represented in Figure 5.

## Discussion

As the number of cases undergoing TEVAR has increased, the number of patients requiring open conversion surgery has for various reasons increased recently. As shown in the study, various pathologies require open conversion, and surgical strategies substantially depend in these situations. Other causes, besides those listed above, such as distal stent graft induced new entry and stent fracture, could be resolved with additional TEVAR; therefore, open conversion was not required in some cases. The actual incidence of open conversion operations in this study is unknown because some patients had undergone previous TEVAR at other institutions.

One of the major reasons for open conversion in proximal aortic lesions is type Ia endoleak. It may be caused by an inherent suboptimal proximal sealing zone (short neck or steep arch angulation) or by dilatation of the proximal aorta during follow up. Another serious complication requiring open aortic arch conversion surgery is RTAD, and its incidence has been reported to be 1–2%.^9^, ^10^, ^11^, ^12^ Regarding RTAD, some cases have been reported to have developed during the TEVAR procedure; however, the two cases in the study occurred in the late period (four and eighty two months later, respectively). On reviewing the three RTAD cases, the diameter of the stent grafts used was found to be oversized from 11% to 15%, given the aortic diameter of the proximal landing zone. In all cases, the oversize ratio exceeded 10%, which may be related to RTAD occurrence. Additionally, it has been reported that devices with proximal bare stents have a higher incidence of RTAD than covered web stents: however, RTAD also occurred in cases with proximal covered web stents. Intra-operative findings revealed an intimal tear approximately 10 mm away from the proximal edge of the stent graft. Although it may be speculation, it is possible that not only direct injury at the edge of the stent graft but also shear stress between the aortic wall firmly fixed by the stent and the proximal native aortic wall pulsating with the cardiac output may lead to the development of intimal tears. In some cases, emergency open aortic arch repair, including entry resection, is required. Regarding the results of open conversion surgery, Joo *et al.* reported that cases involving the aortic arch have worse outcomes; they attributed embolic stroke to DHCA through left thoracotomy in aortic arch surgery.^6^ Recently, the surgical outcomes of open aortic arch surgery through median sternotomy have improved with the widespread use of ASCP.^13^, ^14^, ^15^ All cases of RTAD and type Ia endoleak were treated with median sternotomy arch procedures, and the surgical outcomes in this group have been favourable.

For false lumen enlargement in chronic aortic dissection, thoraco-abdominal aortic surgery, including reconstruction of the visceral branch, is often required. In such cases, minimising the extent of graft replacement is usually possible while preserving the previous stent graft. In these cases, partial extracorporeal circulation is used to maintain perfusion to the lower body and visceral organs, while the aortic wall is clamped with a stent graft to perform distal aortic surgery. There is a concern that this group of patients may require extensive aortic replacement, including previous TEVAR. The extent of aneurysmal disease is reported to be a strong factor associated with SCI.^16^ In the study, paraplegia occurred in two of the four patients with SCI. One of the patients had previously undergone total arch replacement and TEVAR, and underwent open TAAA replacement for further rapid enlargement of the distal region of the stent graft. The other patient had undergone arch replacement, TEVAR, and type IV TAAA replacement. However, despite additional TEVAR, further expansion of the stent graft segment of the descending aortic aneurysm was observed, and the patient underwent open descending aortic replacement and removal of the stent grafts as open conversion surgery for type V endoleak. Both of the paraplegia cases were patients who underwent extensive aortic repair with multiple procedures, and the risk of SCI remains high in such patients. Additionally, in this series, of the 13 patients who underwent DTA or TAAA replacement surgery, pre-operative CSFD placement was not possible in six patients with infection and in three patients on antithrombotic therapy. Thus, two patients with paraplegia were both on antithrombotic therapy, and were not able to place a CSFD tube pre-operatively because urgent surgery did not allow a sufficient cessation period for the antithrombotic drugs. Therefore, CSFD was started after SCI was suspected post-operatively, but did not improve.

The early mortality rate of patients who have undergone open conversion after TEVAR is reported to vary from 6% to 20%.^2^^,^^4^, ^5^, ^6^^,^^17^, ^18^, ^19^, ^20^, ^21^ By comparison, in the patient group it was acceptable at 10%. However, both the early deaths were patients with an AEF; therefore, the strategy for these infected cases remains a critical issue.^20^^,^^22^, ^23^, ^24^, ^25^ A report from an European registry on AEF after TEVAR showed that a conservative approach resulted in a one year mortality rate of 100%, and that the one year survival rate for an oesophageal stenting only approach was 17%. Survival after isolated oesophagectomy was 43%, and it has been reported that aggressive treatment, including radical oesophagectomy, removal of the stent graft, and new aortic reconstruction, contributed to improved survival.^22^ Furthermore, Akashi *et al.* reported from a Japanese multicentre study that omental wrapping is considered effective for AEF.^23^ Therefore, it is also believed that radical strategies, such as oesophagectomy, removal of the infected stent graft, and aortic reconstruction are mandatory for secondary AEF after TEVAR if the patient is likely to tolerate them. However, these radical procedures were highly invasive, and the surgical results were unsatisfactory. Based on limited experience, it was recently thought that the key to improving surgical outcomes for patients with AEF may be early oesophageal reconstruction and establishment of a nutritional programme. In any case, further efforts to improve surgical outcomes for infected cases, including AEF, will continue to be essential.

### Limitations

This study has several limitations. First, this study was performed at a single centre; therefore, only patients who underwent open conversion surgery but not all patients who received TEVAR at the centre were reviewed. Even if complications occur after TEVAR, some patients may be followed medically because of their poor condition. Furthermore, the incidence of open conversion in patients who underwent TEVAR cannot be calculated because open conversion surgery in patients who underwent TEVAR at other hospitals was also included in this study. Second, the number of patients in this study was small. Therefore, conducting statistical analyses of the outcomes is difficult.

### Conclusion

When open conversion surgery is required after TEVAR, the indications are complex, are often associated with infective pathology, and are necessarily high risk particularly in patients with AEF. Previous stent grafts may be preserved in some cases; therefore, endograft management during secondary open surgical conversion after TEVAR presents a specific problem that should be addressed with a tailored approach.

## Funding

None.

## Conflict of interest

None.
